# Neonicotinoid-Contaminated Puddles of Water Represent a Risk of Intoxication for Honey Bees

**DOI:** 10.1371/journal.pone.0108443

**Published:** 2014-12-01

**Authors:** Olivier Samson-Robert, Geneviève Labrie, Madeleine Chagnon, Valérie Fournier

**Affiliations:** 1 Centre de recherche en horticulture, Université Laval, Québec City, Québec, Canada; 2 CÉROM, Centre de recherche sur les grains Inc., Saint-Mathieu-de-Beloeil, Québec, Canada; 3 Département des Sciences Biologiques, Université du Québec à Montréal, Montréal, Québec, Canada; Northwest Fisheries Science Center, NOAA Fisheries, United States of America

## Abstract

In recent years, populations of honey bees and other pollinators have been reported to be in decline worldwide. A number of stressors have been identified as potential contributing factors, including the extensive prophylactic use of neonicotinoid insecticides, which are highly toxic to bees, in agriculture. While multiple routes of exposure to these systemic insecticides have been documented for honey bees, contamination from puddle water has not been investigated. In this study, we used a multi-residue method based on LC-MS/MS to analyze samples of puddle water taken in the field during the planting of treated corn and one month later. If honey bees were to collect and drink water from these puddles, our results showed that they would be exposed to various agricultural pesticides. All water samples collected from corn fields were contaminated with at least one neonicotinoid compound, although most contained more than one systemic insecticide. Concentrations of neonicotinoids were higher in early spring, indicating that emission and drifting of contaminated dust during sowing raises contamination levels of puddles. Although the overall average acute risk of drinking water from puddles was relatively low, concentrations of neonicotinoids ranged from 0.01 to 63 µg/L and were sufficient to potentially elicit a wide array of sublethal effects in individuals and colony alike. Our results also suggest that risk assessment of honey bee water resources underestimates the foragers' exposure and consequently miscalculates the risk. In fact, our data shows that honey bees and native pollinators are facing unprecedented cumulative exposure to these insecticides from combined residues in pollen, nectar and water. These findings not only document the impact of this route of exposure for honey bees, they also have implications for the cultivation of a wide variety of crops for which the extensive use of neonicotinoids is currently promoted.

## Introduction

Pollination is a key ecosystem service for both biodiversity and human welfare. Animal-mediated pollination plays a role in the sexual reproduction process of over 90% of the world's angiosperms, thereby sustaining biodiversity and maintaining the integrity of most terrestrial ecosystems [1], [2]. More than 70% of the world's crop production depends to some extent on biotic pollination, which is primarily performed by insects [3], [4]. Pollination by bees also increases seed set and fruit set, size, quality, shelf life and commercial value of a majority of crops [5]–[9].

While bees are by far the most efficient group of insect pollinators, their populations are declining worldwide [10]–[16]. As a result, over the last decade, pollinator health has been an issue of concern for national and international media, decision makers, scientists and the general public. Several factors, alone or in combination, have been investigated and identified as potential contributing causes of pollinator decline [11], [17]–[19]. Among these, exposure to pesticide, especially of the neonicotinoid family, has been of growing concern. Recent studies have demonstrated that the hive products of honey bee colonies located in agricultural environments across Europe and North America have been contaminated by various agricultural chemicals, including neonicotinoids [20]–[23].

Although neonicotinoid insecticides can be applied in various ways (pulverization, soil dressing), in North America, they are mainly used as a seed dressing to protect corn and soybean crops from a broad range of root-feeding and sucking pest species. In fact, virtually every single seed of corn and a third of soybean seeds are coated with these insecticides in the US, totalizing more than 110 million acres of land for 2010 [24], [25]. The neonicotinoid family is comprised of 10 compounds already in use worldwide or pending approval [26], [27], but clothianidin and thiamethoxam, which degrades to the metabolite clothianidin, are the two major active chemical ingredients used to treat corn and soybeans. Both of these compounds are extremely toxic to pollinators. The recognized amount of clothianidin required to kill 50% of an exposed group of adult honey bees (LD50) after 24 hours ranges from 22–44 ng/bee for contact exposure, and about 3 ng/bee for oral toxicity [28]–[30]. Toxicity is similar for thiamethoxam and LD50 for contact ranges from 24–29 ng/bee and is of 4.4 ng/bee for oral exposure [28], [31]. Given the current rate of application of these compounds to corn crops (between 0.25 mg and 1.25 mg/seed), a single kernel of corn contains enough active ingredients to wipe out an entire honey bee colony. Besides their extreme toxicity, neonicotinoid compounds have been shown to bind in an irreversible fashion to nicotinic acetylcholine receptors (nAChRs) in arthropods [32]. As such, even though insects are able to detoxify their metabolism, once a molecule reaches the brain, its effects become permanent.

Bees can come into contact with these systemic compounds in a number of ways. Recent studies have demonstrated that planting neonicotinoid-coated seeds with a pneumatic drilling machine releases particulate matter contaminated with the insecticides into the environment [23], [33]–[39]. Pollinators foraging in fields and flying in the vicinity of planters can be directly exposed to such clouds of contaminated dust. Furthermore, intoxication is likely to result from collecting and consuming pollen and nectar produced by a plant grown from a neonicotinoid-coated seed [23], [40], grown in soils containing neonicotinoids or covered with contaminated dust during planting [23], [41], [42]. These systemic insecticides can also be very persistent, lingering for several months and even accumulating in plant tissues [43].

In addition to collecting nectar and pollen, honey bees also forage actively for water. High residue levels of neonicotinoids have been measured in guttation and dew water [34], [37], [44]–[46]. Collecting and consuming such contaminated water can result in lethal or sublethal effects for honey bees. The presence of water resources in this form depends largely on specific weather and soils conditions. Given their appearance in the early morning for only a short period of time, it is unclear whether bees are likely to drink from these contaminated drops and thus the risk to bees has been questioned [47]. On the other hand, since neonicotinoid insecticides are highly water soluble and can persist for months in aerobic soil conditions (half-life of clothianidin varies from 148–1,155 days) [30] they are likely to be found in surface waters. Recent studies have indeed found residues of neonicotinoid insecticides in irrigation water, rivers and wetlands in concentrations harmful to some aquatic macro-invertebrates [48]–[53]. Consumption of surface water as an exposure route of pesticide contamination for honey bees has recently been pointed [54]. Nonetheless, lack of data regarding this route of exposure has been underlined by the European Food Safety Authority [55], [56].

This study was initiated after noticing how abundant puddles of water were in corn fields following rainfall and anecdotal observations of honey bees drinking from common puddles of rainwater (albeit not from corn fields). The objectives were to 1) examine whether puddles of water from corn fields are contaminated with neonicotinoid compounds and 2) determine the risk associated with the consumption of this water for honey bees. Considering the extent to which these insecticides are used and their remarkably high toxicity, it is essential to thoroughly understand every potential route by which honey bees can be exposed to them.

## Materials and Methods

### Ethics Statement

No ethics approval was required. We obtained private landowners' permission. Private landowners who granted access in this study wish to remain anonymous and specific GPS coordinates cannot be provided as part of that confidentiality. This study did not involve endangered or protected species.

### Study Area

Sampling was conducted in two neighbouring administrative regions in southern Quebec, Canada. Both regions, Montérégie (45° 37′ 10″ N, 72° 57′ 30″ W) and Estrie (45° 24′ 00″ N, 71° 53′ 03″ W), have historically had high levels of agricultural land use. Montérégie alone produces nearly 60% of the province's corn and soybean crops. Since 2008, close to 100% of corn and over two-thirds of soybean crops have been treated with a neonicotinoid coating. Estrie, on the other hand, produces very little corn and soybean, and its agricultural profile is more evenly distributed among a variety of crops whose seeds are generally untreated with neonicotinoids.

### Field water puddles

Water samples were obtained from puddles of water that had accumulated on the surface of fields following a day of precipitation. All puddles were located at a maximum distance of 1 km from a commercial apiary, well within a honey bee's flight range. In Montérégie, sampling was limited to puddles in corn fields due to the ubiquity of neonicotinoid seed treatment in this crop. Control water samples were collected from puddles in hay fields and grasslands in Estrie and were located at least 3 km from neonicotinoid-coated crops to limit contamination apart and were sampled only once during this study. On June 5^th^, 2012, 10 samples of water were collected from coated corn fields as corn sowing was still in progress. On May 22^nd^, 2013, 30 samples were retrieved during corn plantation, half from coated corn fields and half from hay fields and grasslands. An additional 34 water samples were collected from coated corn fields on June 29^th^, 2013, a full month after sowing had ended. A total of 74 water samples were collected, 15 from untreated crop fields, and 59 from neonicotinoid-treated corn fields. Samples were obtained by collecting water with 50 ml disposable Falcon tubes and filling 1 L amber-coloured glass bottles. Samples were collected from clear water puddles (no suspended solid matter) and tubes were carefully submerged into the puddles to avoid suspending soil particles and to limit sample contamination. Bottles were sealed with aluminum foil-lined lids and immediately placed in a dark cooler. Bottles were stored at 4°C until extraction for chemical analyses, which were done within one week of receiving. Residue analyses were performed by two governmental ISO 17025 accredited laboratories (MAPAQ, CEAEQ).

### Chemical analyses

Water samples collected during corn sowing were analyzed using a modified version of the QuEChERS method originally described by Anastassiades et al. (2003) [57]. Briefly, 60 µl of methanol and 20 µl of isoprocarb standard solution (10 mg/l) were added to 1 ml of each initial water sample. The solution was then filtered through a 0.45 µm PTFE filter, and 10 µl were analyzed by liquid chromatography/mass spectrometry using Waters Acquity LC interfaced to a Waters Xevo TQ MS (Halo C-18 columns, 4.6 solid core x 50 mm porous outer shell with 2.7 µm particle). The mass spectrometer was positioned in a positive electrospray mode and utilized a different MS/MS scan for each pesticide monitored. Liquid chromatography injections were carried out three times. Parent pesticides and metabolites were identified based on comparisons of their chromatographic retention time with known standards and mass abundance ratios to at least two fragment transitions. Ion ratios between the two transitions had to comply with a maximum difference of 20% with the calibration standard. This multi-residue method allows detection of over 400 agrochemical compounds at parts per billion concentration levels. As concentrations of neonicotinoids were expected to drop after corn planting, the analytical method was further modified to include a pre-concentration of the water samples. In brief, 50 µl of isoprocarb standard solution (1 mg/l) and 100 µl of extraction standard were added to 500 ml of each post corn planting water sample. Prepared samples were then passed through Sep-Pak C18 SPE cartridges (1 g, 6 ml), pre-conditioned with 6 ml of methanol and 6 ml of de-ionized water. Cartridges were evaporated to complete dryness under argon gas and then extracted with 2 ml of eluting solution (208 µl of chloridric acid 0.01 N, 25 µl of diethilamine 0.01% in 250 ml of methanol). Extracted cartridges were again evaporation under argon gas to near dryness and extracts were reconstituted in 50 µl of the internal standard and 450 µl of de-ionized water solution (containing 0.1% of formic acid and 5% acetonitrile) for chemical analysis. Original samples consisted of 500 ml and were reconstituted in a 0.5 ml solution thus increasing residue concentrations within the initial sample by a 1000 times. LC-MS/MS analyses were completed using the same analytical method as previously described. These focused analyses were limited to the detection of neonicotinoid pesticides and other pesticides intensively used in Quebec province and commonly encountered in water in agricultural areas at parts per trillion concentration levels.

### Conversions and risk evaluation

Chemical analyses of water result in concentrations expressed in mass of active ingredient per volume of water. In order to understand the potential exposure for bees, the amount of water a honey bee would consume on a daily basis and thus the amount of pesticide it would ingest must be estimated. The drinking water intake rate used in this risk assessment method is based on direct measurement of the water flux rate of the brown paper wasp (*Polistes fuscatus*). The brown paper wasp and the honey bee are taxonomically related (both of the Hymenoptera order), share similar size and weight and are both social species that utilize water for thermoregulation of their nest [58]. Furthermore, this drinking water intake rate accounts for all sources of water intake (primarily food and drink). As reported by the US EPA's White Paper in Support of the Proposed Risk Assessment Process for Bees (2012) [59], a worker bee must drink a maximum of 0.047 ml of water per day in order to satisfy its daily metabolic water needs. The process for determining risk to honey bees is based on a Risk Quotient (RQ), and is consistent with the process used for other taxa [54]. RQ is expressed as the ratio of point estimates of dietary exposure, in this case, the drinking water intake rate, to point estimates of effects, as established by the acute oral lethal dose to 50% of the organisms tested (LD50). For example, considering clothianidin's LD50 at 24 hours is of 3.35 ng/bee and a honey bee would ingested in a day 2.5 ng of clothianidin through pollen, nectar or water consumption, then the corresponding RQ value would be of 0.75 (2.5/3.35) In consideration of the historic average dose response relationship for acute toxicity studies with bees, the acceptable limit of the acute RQ value was set below 0.4 [59]. An acute RQ value of 0.4 or higher should raise concerns.

## Results

### Multiresidue analyses of puddle water

Chemical analyses of puddle water indicated that honey bees are exposed to various agricultural chemicals through collection and consumption of water. A total of 30 different pesticides and metabolites were found in the 74 puddle water samples, with an average of 3.9±2.6 chemicals detected per sample. In the 15 control water samples (untreated-crop fields), 5 pesticides were identified, with some samples containing all 5 and an average of 2.1±3.8 chemicals per sample, always below the limit of quantification. Of the 5 pesticides detected, 4 were herbicides (atrazine, desethylatrazine, metolachlore and simazine) and 1 was a fungicide (thiabendazole). Since occurrence and concentrations of neonicotinoids were similar in water samples collected from corn fields when corn was still being sown, samples from 2012 (10) and 2013 (15) were pooled together in **Table 1**. Also, the diversity of pesticides found in these puddles was similar for both years, with the exception of metolachor, which was ubiquitous in 2012 and identified only once in 2013. In these 25 water samples collected in both years (Table 1), 22 pesticides and metabolites were identified, with an average of 6.4±2.6 chemicals detected per sample and up to 14 different compounds in a single sample. Neonicotinoid concentrations ranged from 0.1 to 55.7 µg/l (ppb) for clothianidin and from 0.1 to 63.4 µg/l for thiamethoxam. In the 34 water samples collected from corn fields one month after planting was completed (**Table 2**), 10 pesticides and degradation products were identified, with an average of 2.8±0.6 agrochemicals per sample and up to 4 compounds per sample. Concentrations of neonictoinoid compounds ranged from 0.017 to 2.3 µg/l for clothianidin, from 0.004 to 2.8 µg/l for thiamethoxam and from 0.001 to 0.007 µg/l for imidacloprid. Most of the pesticides found one month after planting were identified at concentrations under the limit of quantification, with the exception of azoxystrobin, clothianidin and thiamethoxam. All water samples collected from corn fields contained residues of at least one neonicotinoid insecticide, and 83% of these samples contained residues of both clothianidin and thiamethoxam.

**Table 1:** Pesticide concentrations found in puddle water samples taken from a corn field in 2012 and 2013, when planting was in progress.

Pesticide	Class*	Detection	Samples (N)	%	Concentrations (µg/L)				LOQ†
					Min	Max	Mean‡	SEM‡	
Atrazine	HERB, S	25	25	100	0.1	7189.0	312.8	1434.6	0.1
Thiabendazole	FUNG, S	25	25	100	0.1	5.7	0.6	1.3	0.1
Clothianidin	NEO, S	23	25	92	0.1	55.7	4.6	12.1	0.1
Desethylatrazin	HERB	21	25	84	0.1	705.0	39.5	152.9	0.1
Thiamethoxam	NEO, S	18	25	72	0.1	63.4	7.7	16.7	0.1
Metolachlor	HERB, PS	11	25	44	0.2	10660.0	1401.9	3353.9	0.1
Metalaxyl	FUNG, S	10	25	40	0.1	0.7	0.4	0.2	0.1
Propazine	HERB	7	25	28	0.4	170.7	25.1	64.2	0.1
Spiroxamine	FUNG	5	25	20	0.4	49.5	13.9	20.1	0.1
Mesotrione	HERB	4	25	16	9.7	10681.0	3437.6	5036.5	0.1
Imazethapyr	HERB	3	25	12	0.1	1.6	0.6	0.8	0.1
Boscalid	FUNG, S	2	25	8	0.2	0.8	0.5	0.4	0.1
Dimetachlore	HERB	2	25	8	3.5	7.1	5.3	2.5	0.1
Dimethenamid	HERB	2	25	8	0.1	0.1	0.1	0.0	0.1
Simazine	HERB, S	2	25	8	1.3	40.7	21.0	27.9	0.1
Benoxacor	HEBR	1	25	4	6.1	6.1	6.1	NA	0.1
Bentazone	HERB	1	25	4	1.5	1.5	1.5	NA	0.1
Chlorimuron-ethyle	HERB	1	25	4	0.4	0.4	0.4	NA	0.1
Metobromuron	HERB	1	25	4	1.5	1.5	1.5	NA	0.1
Nicosulfuron	HERB, S	1	25	4	8.4	8.4	8.4	NA	0.1
Picoxystrobin	FUNG	1	25	4	2.5	2.5	2.5	NA	0.1
Rimsulfuron	HERB	1	25	4	6.0	6.0	6.0	NA	0.1

* Class: FUNG  =  fungicide, HERB  =  herbicide, NEO  =  neonicotinoid, PS  =  partially systemic, S =  systemic.

† LOQ  =  limit of quantification (µg/L).

‡ Mean and SEM for detections> LOQ.

**Table 2:** Pesticide concentrations found in puddle water samples taken from a corn field one month after planting was completed (in 2013).

Pesticide	Class*	Detection	Samples (N)	Proportion of	Concentrations (µg/L)				LOQ†
				positives (%)	Min	Max	Mean‡	SEM‡	
Clothianidin	NEO, S	34	34	100.0	0.0170	2.3000	0.523	0.567	0.001
									
Thiamethoxam	NEO, S	34	34	100.0	0.004	2.8	0.585	0.632	0.0001
									
Azoxystrobin	FUNG, S	21	34	61.8	0.001	2.1	0.191	0.587	0.001
Imidacloprid	NEO, S	3	34	8.8	0.001	0.007	0.004	0.003	0.001
Imidacloprid urea	NEO, S	3	34	8.8	0.005	0.005	0.005	0	0.0009

* Class: FUNG  =  fungicide, HERB  =  herbicide, NEO  =  neonicotinoid, PS  =  partially systemic, S =  systemic.

† LOQ  =  limit of quantification (µg/L).

‡ Mean and SEM for detections> LOQ.

### Risk assessment of neonicotinoid insecticides in water

Comparison of mean concentrations of clothianidin and thiamethoxam potentially ingested per honey bee with their respective oral LD50 values revealed a mean acute risk quotient (RQ) below 0.1 for samples collected during corn planting (**Table 3**). However, comparisons of the maximum concentrations per bee with the LD50 values show acute risk quotients of 0.78 and 0.68 for clothianidin and thiamethoxam respectively, above the accepted level of concern of 0.4 determined by historical risk assessment. For water samples collected one month after corn planting, comparison of mean concentrations per bee with their respective oral LD50 values indicates a mean risk quotient of 0.01. No puddle of water contained neonicotinoid compounds in concentrations at or above a lethal dose.

**Table 3:** Risk assessment of puddle water during corn planting and one month after completion (2012–2013).

Neonicotinoid	AOT LD50 (ng/bee)*	Planting	Samples (N)	Concentrations in water (µg/L)		Body burden in bees (ng/bee)†		RQ‡	
				Mean§	Max	Mean§	Max	Mean§	Max
Clothianidin	3.35	During	25	4.6	55.7	0. 21	2.62	0.06	0.78
		After	34	0.5	2.3	0. 02	0. 11	0.01	0.03
Thiamethoxam	4.4	During	25	7.7	63.4	0. 36	2.98	0.08	0.68
		After	34	0.6	2.8	0. 03	0. 13	0.01	0.03

* Acute oral toxicity (AOT) values at 24 hours [83].

† Conversions are based on the drinking water intake rate of 0.047 ml (EFED & PMRA 2012).

‡ RQ  =  Risk Quotient.

§ Mean for detections> LOQ.

## Discussion

Neonicotinoid seed dressing is used extensively in agriculture to protect a wide variety of crops from pests. As these insecticides are highly toxic to honey bees, it is essential to identify and quantify every potential route of exposure. Field observations of honey bees drinking from puddles of rainwater raised concerns about their potential exposure to these systemic compounds.

### Neonicotinoid residues in puddles of water

The results presented here more clearly define a previously uninvestigated route by which honey bees are exposed in corn-dominated environment, not only to neonicotinoid insecticides, but also to a cocktail of herbicides and fungicides (**Table 1** and **2**). Not surprisingly, neonicotinoids were the only insecticidal compounds detected in all samples, due to their water solubility. Concentrations of neonicotinoid residues in puddles were markedly higher in springtime (mid-May) than at the beginning of summer (end of June). This would indicate that much of the residue in these puddles is the result of drifting and deposition of contaminated dust emitted during sowing of neonicotinoid-coated seeds. Recent studies have found extremely high levels of clothianidin and thiamethoxam in planter exhaust material and in the vicinity of the planter itself [23], [34], [35], [37]. This airborne particulate matter is highly susceptible to drifting, settling and thereby contaminating the soil surface and nearby water bodies. Precipitation can readily dissolve neonicotinoid compounds in the superficial layer of soil, and they remain in the rainwater puddles. However, the soil itself represents an even greater source of puddle contamination. For purposes of comparison, the aerial dust emitted during sowing actually comprises less than 2% of the total amount of active ingredients in seed dressing, whereas the remaining 78–96% of active ingredients surrounding the seeds are not absorbed by the plant and enter the soil [60]. Given the particularly persistent nature of neonicotinoids combined with repeated applications over successive years, accumulating concentrations in soils can be expected [61], [62]. The amounts of neonicotinoids present in soil play an important role in the contamination of water puddles.

### Implications and flaws of risk assessment

While the average acute risk associated with consumption of puddle water alone was found to be relatively low for pollinators (**Table 3**), some puddles contained levels of neonicotinoids almost as high as the LD50 for honey bees, and the risk associated with consumption of this water is high. Although average concentrations of neonicotinoids per bee exposed to contaminated puddle water were under lethal doses, these levels are nonetheless sufficiently high to elicit various sublethal effects at both the individual and colony levels. Sublethal effects include increased viral replication (from 0.0001 ppb, [63]), reduced food consumption (from 0.001 ppb, [64]), reduced fecundity (from 0.001 ppb, [65]), decreased size of hypopharyngeal glands (from 0.002 ppb, [66]), impaired foraging behaviour (from 0.0038 ppb, [67]) and reduced colony growth and queen production (0.007 ppb, [68]).

Risk assessment for contact with and dietary exposure to pesticides is a process thoroughly described for honey bees [54], but the risk associated with water has been only minimally investigated, as water is often perceived as a less important resource for pollinators. Current risk assessment draws an incomplete portrait of the situation. First, risk assessments evaluate the danger of a single pesticide compound at a time, whereas most of our water samples contained measurable residue of both clothianidin and thiamethoxam. Since these two compounds belong to two different structural types and exhibit non-competitive binding to nicotinic acetylcholine receptors in insects [69], [70], the risk associated with the consumption of such water is underestimated. A comprehensive risk assessment should consider residues of clothianidin and thiamethoxam additively as they act independently of each other and their combined effect is, therefore, the sum of their individual effects. Secondly, social insects such as the honey bee need to consume water for metabolic reasons, but will mostly transport it back to the hive. Studies have demonstrated that the honey stomach is permeable to some pesticides [71]–[73] and active neonicotinoid ingredients can therefore penetrate through the foregut cuticle in the same way as the leaf cuticle [74]. As such, complete consumption is not necessary in order for these compounds to enter the hemolymph of the insect. Honey bees require a considerable volume of water for nest-related tasks such as the dilution of stored honey to feed the brood, to maintain humidity in the colony for larval and pupal development and for evaporative cooling to thermoregulate the nest [75]–[78]. Honey bees are known to make 50–100 trips to forage for water every day [76], [79]. During each of these trips, they will generally collect 0.030–0.060 ml of liquid [47], [79], [80]. As a result, a forager is estimated to collect 1.5–6 ml of water per day [59]. Although metabolic needs are small and the vast majority of collected water will be regurgitated once inside the hive, a certain amount of pesticide will cross the gut wall during transportation thus exposing the honey bee to these pesticides. However, to our knowledge, the gut wall penetration rate for neonicotinoids is currently unkown. Taking these varied water needs into account, a comprehensive estimate of water collected is much greater than the estimated drinking water intake rate of 0.047 ml used in evaluating the risk associated with a contaminated water supply. Risk assessment based solely on the daily drinking water intake rate vastly underestimates pesticide exposure. Furthermore, the real dietary risk to bees is not only limited to water resources but also has to consider collection and consumption of contaminated pollen and nectar as frequent, daily routes of exposure to all pesticide residues, whether they are systemic or not.

### Occurrence of water puddles and relative importance to honey bees

Water collection depends entirely upon the colony's demand, since water is not stored inside the hive [78]. As such, water carriers will collect water in the immediate environment of the colony. Since water puddles are extremely abundant at the surface of fields after precipitation and lie well within a honey bee flight range, they are very likely to exploit this water supply. Paradoxically, it seems that honey bee foragers become increasingly motivated to collect water after being confined inside the hive by cool, rainy weather [75], [77], which is precisely when water puddles are most abundant. Moreover, honey bees are not naturally inclined to collect clean water, but rather prefer more natural, stagnant bodies of water containing organic matter and minerals [81]. Water temperature is also an important factor, as honey bees prefer to collect water from a warmer source, so as not to impede their flight ability [82]. Puddles of water are naturally heated by the sun, possess a distinct organic and saline “smell” on the surface of agricultural fields and are abundant in the colony's surroundings, all of which make them remarkably attractive to honey bees. One downside of being heated by the sun is the resulting evaporation. Although some pesticides may evaporate along with the water or degrade under warmer conditions, residue concentrations of systemic compounds such as neonicotinoids and herbicides would build up as the water evaporates and thus increase the risk of puddle water in comparison with other surface water.

## Conclusions

To our knowledge, this is the first scientific record of neonicotinoid residues in in-field puddles of water in relation with neonicotinoid seed dressing in corn cropping system. Although concentrations of these systemic insecticides in water samples were not found to be above lethal doses, repeated exposure through consumption of puddle water alone can result in various sublethal effects at the individual- and colony-level. Moreover, due to the abundance of water puddles in agriculture-intensive areas and their particularly attractive features for honey bees, they are highly likely to be one of the main, and at times exclusive, supply of water and thus an important source of pesticide exposure Finally, we believe that the risk of exposure to neonicotinoid-contaminated water reported here is an underestimation. Additional, comprehensive research is needed to therefore better assess risk associated with water use for honey bees. Our findings provide further evidence of the widespread environmental contamination with neonicotinoids and highlight another potential route of exposure for honey bees and other pollinators.

## Supporting Information

Data S1 **Information file for Data S2.**
(DOCX)

Data S2 **Data on the 74 water sample analyses used in the experiment.**
(CSV)
