# Correction: Neonicotinoid-Contaminated Puddles of Water Represent a Risk of Intoxication for Honey Bees

**DOI:** 10.1371/journal.pone.0119357

**Published:** 2015-03-23

**Authors:** The PLOS ONE Staff

There are errors in the Supporting Information. In Dataset S1, the unit of measurement is incorrectly labeled. The corrected label is: “(Concentrations, in μg/L)”.

In Dataset S2, the values of 0.7, 0.8 and 0.9 are incorrect. The corrected values are 0.0007, 0.0008 and 0.0009.

Please view the corrected Datasets S1–2 below.

## Supporting Information

S1 Data Information file for S2 Data. (DOCX)

S2 Data Data on the 74 water sample analyses used in the experiment. (CSV)
