# Supplementary material for: Neonicotinoid-Contaminated Puddles of Water Represent a Risk of Intoxication for Honey Bees
Source: PLoS One. 2014 Dec 1;9(12):e108443. doi: 10.1371/journal.pone.0108443 (PMC4249843; doi:10.1371/journal.pone.0108443)
Supplement: Data S1 — Information file for Data S2. (DOCX) [file pone.0108443.s001.docx]

Filename : Samson-Robert, Labrie, Chagnon, Fournier (2014) Data from “Neonicotinoid-Contaminated Puddles of Water Represent a Risk of Intoxication for Honey Bees”.csv.

This file include data on the 74 water sample analyses used in the experiment published in Neonicotinoid-Contaminated Puddles of Water Represent a Risk of Intoxication for Honey Bees (2014) Samson-Robert, O., Labrie, G., Chagnon, M. & Fournier, V. PLoS ONE.

The data was collected in the field by O. Samson-Robert. Please contact Prof. V. Fournier if you have any questions (valerie.fournier@fsaa.ulaval.ca).

The file contains the following data columns :

Sample (Individual laboratory identification number)

Treatment (Exposed, Control)

Date (Date at which samples were collected, May-22, June-05, June-29)

Year (2012, 2013)

Period (Period of time relative to corn sowing, During or After)

Acetamiprid (Concentrations, in ppb)

Atrazine (Concentrations, in ppb)

Azoxystrobin (Concentrations, in ppb)

Benoxacor (Concentrations, in ppb)

Bentazone (Concentrations, in ppb)

Boscalide (Concentrations, in ppb)

Chlorimuron-ethyle (Concentrations, in ppb)

Clothianidin (Concentrations, in ppb)

Desethylatrazin (Concentrations, in ppb)

Dimetachlore (Concentrations, in ppb)

Dimethenamid (Concentrations, in ppb)

Fenamidone (Concentrations, in ppb)

Fenamidone.metabolite (Concentrations, in ppb)

Imidacloprid (Concentrations, in ppb)

Imidacloprid guanidine (Concentrations, in ppb)

Imidacloprid olefin (Concentrations, in ppb)

Imidacloprid urea (Concentrations, in ppb)

Imazethapyr (Concentrations, in ppb)

Mesotrione (Concentrations, in ppb)

Metalaxyl (Concentrations, in ppb)

Metobromuron (Concentrations, in ppb)

Metolachlor (Concentrations, in ppb)

Nicosulfuron (Concentrations, in ppb)

Picoxystrobin (Concentrations, in ppb)

Propazine (Concentrations, in ppb)

Rimsulfuron (Concentrations, in ppb)

Simazine (Concentrations, in ppb)

Spiroxamine (Concentrations, in ppb)

Thiabendazole (Concentrations, in ppb)

Thiamethoxam (Concentrations, in ppb)

NA is used to indicate missing data.
