# Supplementary material for: Correction: Neonicotinoid-Contaminated Puddles of Water Represent a Risk of Intoxication for Honey Bees
Source: PLoS One. 2015 Mar 23;10(3):e0119357. doi: 10.1371/journal.pone.0119357 (PMC4370396; doi:10.1371/journal.pone.0119357)
Supplement: S1 Data — (DOCX) [file pone.0119357.s001.docx]

Filename : Samson-Robert, Labrie, Chagnon, Fournier (2014) Data from “Neonicotinoid-Contaminated Puddles of Water Represent a Risk of Intoxication for Honey Bees”.csv.

This file include data on the 74 water sample analyses used in the experiment published in Neonicotinoid-Contaminated Puddles of Water Represent a Risk of Intoxication for Honey Bees (2014) Samson-Robert, O., Labrie, G., Chagnon, M. & Fournier, V. PLoS ONE.

The data was collected in the field by O. Samson-Robert. Please contact Prof. V. Fournier if you have any questions (valerie.fournier@fsaa.ulaval.ca).

The file contains the following data columns :

Sample (Individual laboratory identification number)

Treatment (Exposed, Control)

Date (Date at which samples were collected, May-22, June-05, June-29)

Year (2012, 2013)

Period (Period of time relative to corn sowing, During or After)

Acetamiprid (Concentrations, in μg/L)

Atrazine (Concentrations, in μg/L)

Azoxystrobin (Concentrations, in μg/L)

Benoxacor (Concentrations, in μg/L)

Bentazone (Concentrations, in μg/L)

Boscalide (Concentrations, in μg/L)

Chlorimuron-ethyle (Concentrations, in μg/L)

Clothianidin (Concentrations, in μg/L)

Desethylatrazin (Concentrations, in μg/L)

Dimetachlore (Concentrations, in μg/L)

Dimethenamid (Concentrations, in μg/L)

Fenamidone (Concentrations, in μg/L)

Fenamidone.metabolite (Concentrations, in μg/L)

Imidacloprid (Concentrations, in μg/L)

Imidacloprid guanidine (Concentrations, in μg/L)

Imidacloprid olefin (Concentrations, in μg/L)

Imidacloprid urea (Concentrations, in μg/L)

Imazethapyr (Concentrations, in ppb μg/L

Mesotrione (Concentrations, in μg/L)

Metalaxyl (Concentrations, in μg/L)

Metobromuron (Concentrations, in μg/L)

Metolachlor (Concentrations, in μg/L)

Nicosulfuron (Concentrations, in μg/L)

Picoxystrobin (Concentrations, in μg/L)

Propazine (Concentrations, in μg/L)

Rimsulfuron (Concentrations, in μg/L)

Simazine (Concentrations, in μg/L)

Spiroxamine (Concentrations, in μg/L)

Thiabendazole (Concentrations, in μg/L)

Thiamethoxam (Concentrations, in μg/L)

NA is used to indicate missing data.
